# Influence of the Hubbard U Correction on the Electronic Properties and Chemical Bands of the Cubic (*P*m$\bar{3}$m) Phase of SrTiO_3_ Using GGA/PBE and LDA/CA-PZ Approximations

**DOI:** 10.3390/molecules29133081

**Published:** 2024-06-28

**Authors:** Issam Derkaoui, Mohamed Achehboune, Roberts I. Eglitis, Anatoli I. Popov, Issam Boukhoubza, Mohamed A. Basyooni-M. Kabatas, Abdellah Rezzouk

**Affiliations:** 1 Laboratory of Solid State Physics, Faculty of Sciences Dhar el Mahraz, University Sidi Mohammed Ben Abdellah, P.O. Box 1796, Atlas Fez 30 000, Morocco; derkaouiissam@gmail.com (I.D.);; 2 Laboratoire de Physique des Solides, Namur Institute of Structured Matter, University of Namur, Rue de Bruxelles 61, 5000 Namur, Belgium; 3 Institute of Solid State Physics, University of Latvia, 8 Kengaraga Str., LV1063 Riga, Latvia; 4 Dynamics of Micro and Nano Systems Group, Department of Precision and Microsystems Engineering, Delft University of Technology, Mekelweg 2, 2628 CD Delft, The Netherlands; 5 Department of Nanotechnology and Advanced Materials, Graduate School of Applied and Natural Science, Selçuk University, Konya 42030, Turkey; 6 Solar Research Laboratory, Solar and Space Research Department, National Research Institute of Astronomy and Geophysics, Cairo 11421, Egypt

**Keywords:** STO, DFT, Hubbard U, electronic properties, band gaps, chemical bonds

## Abstract

By using DFT simulations employing the GGA/PBE and LDA/CA-PZ approximations, the effects of the Hubbard U correction on the crystal structure, electronic properties, and chemical bands of the cubic phase (*P*m$\bar{3}$m) of STO were investigated. Our findings showed that the cubic phase (*P*m$\bar{3}$m) STO’s band gaps and lattice parameters/volume are in reasonably good accordance with the experimental data, supporting the accuracy of our model. By applying the DFT + U method, we were able to obtain band gaps that were in reasonably good agreement with the most widely used experimental band gaps of the cubic (*P*m$\bar{3}$m) phase of STO, which are 3.20 eV, 3.24 eV, and 3.25 eV. This proves that the Hubbard U correction can overcome the underestimation of the band gaps induced by both GGA/PBE and LDA/CA-PZ approximations. On the other hand, the Sr-O and Ti-O bindings appear predominantly ionic and covalent, respectively, based on the effective valence charges, electron density distribution, and partial density of states analyses. In an attempt to enhance the performance of STO for new applications, these results might also be utilized as theoretical guidance, benefitting from our precise predicted values of the gap energies of the cubic phase (*P*m$\bar{3}$m).

## 1. Introduction

ABO_3_ perovskites represent the most recently researched materials, in which both A and B represent cations of different sizes linked to an oxygen anion. The alkaline earth metal group includes A cations such as Ra, Ba, Sr, Ca, Mg, and Be, which are slightly larger than the transition metal group’s other B cations (i.e., Ti and Zr). A significant perovskite oxide among these ABO_3_ perovskites is strontium titanate (STO), which has received considerable attention in the last few years owing to its extraordinary physical properties, rendering it a promising material suitable for a wide variety of applications, including grain boundary barrier layer capacitors [1], optical switches [2,3] oxygen-gas sensors [4,5], environmental remediation [6,7,8], and solar energy utilization [9,10].

Strontium titanate takes different polytypic crystal forms, where the orthorhombic and tetragonal phases exist at low temperatures. In contrast, the cubic phase occurs at higher temperatures above 300 K, exhibiting the most stable phase among the three crystal forms. Both experimentally [11,12,13,14] and theoretically [15,16,17,18,19,20,21,22], the structural, physical, electronic, and optical properties of the cubic (*P*m$\bar{3}$m) phase of STO have recently been extensively studied. Experimentally, the most common experimental band gaps of the cubic (*P*m$\bar{3}$m) phase of STO are 3.20 eV [11], 3.22 eV [12], and 3.25 eV [13]. Theoretically, the GW methods have been used to investigate the direct and indirect gaps of SrTiO_3_ in great depth [23,24]. In addition, several theoretical works demonstrate that hybrid exchange-correlation functions like B3LYP and B3PW better predict the band-gap values versus the experimental data [16,17,18,19]. However, the classical density function theory (DFT) method, with moderate computational cost, such as the local density approximation (LDA) and generalized gradient approximation (GGA), fails to describe the electronic structure accurately and underestimates the measurements of band-gap values. For example, the band-gap values of the cubic (*P*m$\bar{3}$m) phase of STO computed from the classical DFT methods were found to be 1.90 eV [20], 1.79 eV [21], and 1.73 [22] with LDA, LDA/PW, and GGA approximations, respectively.

To solve this particular challenge and to surmount the limitations of the LDA and GGA computations, it is necessary to add the fitting semi-empirically parameter “Hubbard U potential”, which is affected by the correlated electronic states (“d” and “f” orbitals) by separating a few degrees of freedom, which are relevant for the correlation [25,26,27,28]. Thus, the Hubbard U potential values should be used to move the localized states (“d” and “f” orbitals) away from the Fermi level, and this is performed by adding a term to the Hamiltonian that increases the total energy and prevents unwanted delocalization. In our case, with the application of the Hubbard U potential to the cubic (*P*m$\bar{3}$m) phase of STO, the 3d states of Ti and 4d states of Sr are shifted away from the Fermi level, giving quite good results compared with the experimental values [11,12,13]. 

The primary motivation of this work, based on the correction of the Hubbard U potential, is to give a perfect prediction of the most common experimental band gaps of the cubic phase (*P*m$\bar{3}$m) STO utilizing the GGA/PBE and LDA/CA-PZ approximations. The structure of this work is as follows: Initially, we look for the plane wave pseudopotential methods, k-points, and cut-off energy that provide the optimum lattice parameters/volume with the least amount of divergence (less than 1%) from actual experimental data. Secondly, a thorough investigation of the adopted Hubbard U potential for the Ti-3d, Sr-4d, and O-2p orbitals is given to outline the electronic structures precisely, thus overcoming the underestimation induced by the GGA/PBE and LDA/CA-PZ approximations. Furthermore, the calculated electronic properties and chemical bonding are discussed, followed by conclusions.

## 2. Results and Discussion

### 2.1. Geometry Optimization

Geometrical optimizations were carried out to reduce the external stresses to estimate the structural and electronic characteristics of the STO perovskite material. This makes it possible to relax the STO crystal lattices, obtaining equilibrium lattice parameters and volume in perfect accordance with experimental results. Subsequently, we investigated the most suitable plane wave pseudopotential methods, k-points, and cutoff energies for the cubic (*P*m$\bar{3}$m) phase of STO, using two different DFT approximations (GGA/PBE and LDA/CA-PZ).

#### 2.1.1. Appropriate Pseudopotential Methods, K-Points, and Cut-Off Energy

**Figure 1:**
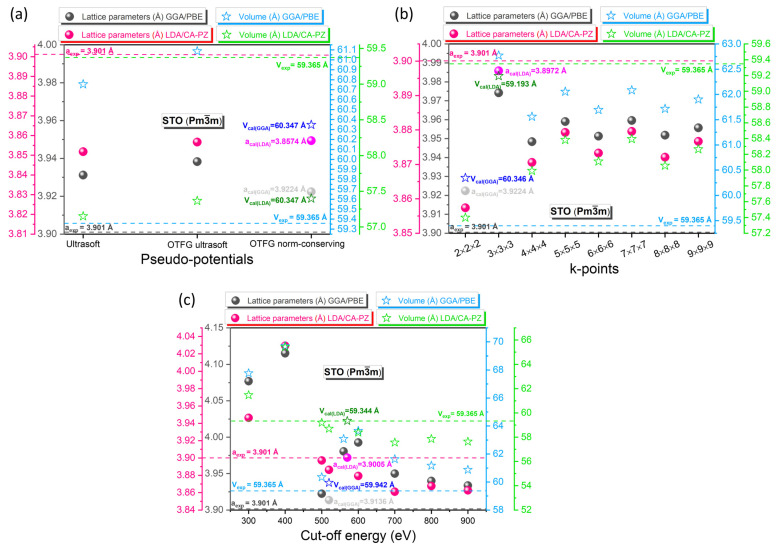
Computation and comparison with the experiment of the cell parameters and volume of the cubic phase of STO utilizing the GGA/PBE and LDA/CA-PZ approximations as a function of (**a**) the pseudopotential methods, (**b**) the k-points values, and (**c**) the cut-off energy values. The k-points and cut-off energy values were fixed at 2 × 2 × 2 and 500 eV to choose the most appropriate pseudopotential methods, respectively. The cut-off energy was set at 500 eV in the computation involving a change in the values of the k-points. To compute with varying cut-off energy values, the k-points values for the GGA/PBE approximation and the LDA/CA-PZ method were set to 2 × 2 × 2 and 3 × 3 × 3, respectively.

Lattice parameters and volume calculated by DFT functionals in this work and compared with the experimental results, as a function of different pseudopotential methods and k-points/cut-off energy values, for the cubic (*P*m$\bar{3}$m) phase of STO are shown in Figure 1. At first, we searched for the most appropriate plane-wave pseudopotential methods (the ultrasoft, the OTGF norm-conserving, and the OTGF ultrasoft) for STO (see Figure 1a). Additionally, Table S1 in the Supplementary Materials Section shows a whole computation of these values and the volume and lattice parameter deviations.

Initially, the primitive lattice parameters a = b = c = 3.901 Å (α=90°; volume = 59.365 Å^3^) [29] were used for constructing the cubic (*P*m$\bar{3}$m) phase of STO. Relative to the same measured experimental data from Abramov et al. [29], the relative deviations from experimental values of the calculated lattice parameters for the GGA/PBE (LDA/CA-PZ) approximation are about 1.097% (−1.277%) for the ultrasoft, 1.004% (−1.148%) for the OTGF ultrasoft, and 0.545% (−1.130%) for the OTGF norm-conserving pseudopotential methods (Figure 1a). For GGA approximation, it should be noted that the OTGF norm-conserving pseudopotential, even if it offers better deviations in the lattice parameters and volume than the OTGF ultrasoft pseudopotential, causes a problem related to the disappearance of the Sr-4d orbital in the density of states. For this reason, we chose the OTGF ultrasoft pseudopotential, and for a better understanding, we present the two partial densities of states of the two potentials (see Figure S1). Therefore, the lattice parameters for STO calculated by OTGF ultrasoft (GGA/PBE) and OTFG norm-conserving (LDA/CA-PZ) pseudopotential methods have the smallest relative deviation from the experimental value.

After finding the most suitable pseudopotential methods for the cubic (*P*m$\bar{3}$m) phase of STO, we then searched for the most suitable k-points and cut-off energy values. As a preliminary step for both the GGA/PBE and LDA/CA-PZ approximations, the energy cut-off value was kept constant (e.g., 500 eV), and the grid values (k-points) were varied (Figure 1b). Once we obtained an appropriate k-point value, we held hod this value constant and modified the cut-off energy to achieve the most appropriate cut-off energy value (Figure 1c). Tables S2 and S3 in the Supplementary Materials Section also calculate the difference between lattice parameters and volume as a function of varying k-points and cut-off energy values. When the k-points are fixed at 2 × 2 × 2 and 3 × 3 × 3, using the GGA/PBE and LDA/CA-PZ approximations, respectively, the divergence from the experimental and optimized volume and lattice parameters is the smallest (Figure 1b coupled with Table S2). Furthermore, we found that after applying several cut-off energies, the minimum deviation of the lattice parameters and volume is 0.730% (−0.012%) and 2.178% (−0.035%) using the GGA/PBE (LDA/CA-PZ) approximation, respectively, and this is for the cut-off energy value of 800 eV (570 eV) under the GGA/PBE (LDA/CA-PZ) approximation (Figure 1c coupled with Table S3). 

**Figure 2:**
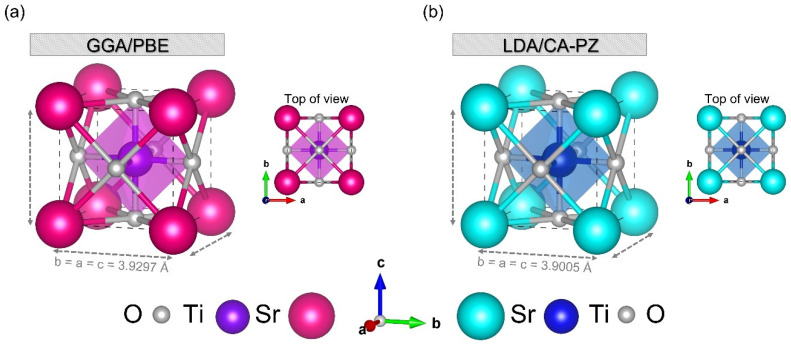
Crystalline structure schematic of the STO cubic phase primitive cell using (**a**) GGA/PBE approximation and (**b**) LDA/CA-PZ method.

Table 1 summarizes the most suitable k-points and cut-off energy values of the cubic (*P*m$\bar{3}$m) phase of STO perovskite material using the GGA/PBE (LDA/CA-PZ) approximation. Furthermore, the primitive cell of the cubic (*P*m$\bar{3}$m) phase of STO with five atoms with local symmetry $O_{h}^{1}$ Figure 2 illustrates that Sr is sitting at the body center, Ti is positioned at the origin (0.0, 0.0, 0.0) a, and oxygen is face-centered, where the optimized lattice parameters computed by GGA/PBE and LDA/CA-PZ approximations are a = b = c = 3.9297 Å (α=90°; volume = 60.687 Å^3^) (Figure 2a) and a = b = c = 3.9005 Å (α=90°; volume = 59.344 Å^3^) (Figure 2b), respectively. These results show that the variation of lattice parameters from calculated and standard values [29] for STO perovskite material was less than 0.74% and 0.013% for the GGA/PBE and LDA/CA-PZ approximations, respectively. The cut-off and k-point values selected as a result of optimization are trustworthy and demonstrate our model’s applicability while exhibiting the lowest relative deviation from the experimental values. In addition, in Table 2, a more extensive comparison between our optimized lattice parameters and those obtained experimentally [29,30] and theoretically [20,31,32] for the cubic (*P*m$\bar{3}$m) of STO is presented.

#### 2.1.2. Hubbard Potential Correction

We used the modified Hubbard U potential for the Sr-4d, Ti-3d, and O-2p orbitals to improve the accuracy of the electronic property calculations and to produce better results in agreement with real data. In fact, the Hubbard potential U is often used as a fitting parameter. The values of U can be adjusted iteratively until a good agreement between theory and experiment is obtained. Effectively, it is found semi-empirically by searching for values, such as the band gap of a particular material, that reproduce the experimental results. By isolating a few degrees of freedom relevant to the correlation, the addition of the Hubbard U potential to the standard DFT approach can be used to characterize very strongly correlated electronic states [25,26,33].

According to the experimental results, different close values of the gap energies of the cubic (*P*m$\bar{3}$m) phase of STO were observed: 3.20 [11], 3.22 [12], and 3.25 [13]. Therefore, a systematic study was conducted by adjusting the band gaps and exploring the Hubbard correction in the case of the two approximations, namely GGA-PBE and LDA/CA-PZ. The band gaps for the cubic (*P*m$\bar{3}$m) phase of STO were determined to be 1.964 eV and 1.715 eV, respectively, in the spin polarization regime and without the Hubbard correction, using the GGA/PBE and LDA/CA-PZ approximations (Table 3). To appropriately represent the electronic structures, the modified Hubbard U potential was thus employed for the Ti-3d, Sr-4d, and O-2p orbitals. This may compensate for the underestimation caused by both the GGA/PBE and LDA/CA-PZ approximations.

**Figure 3:**
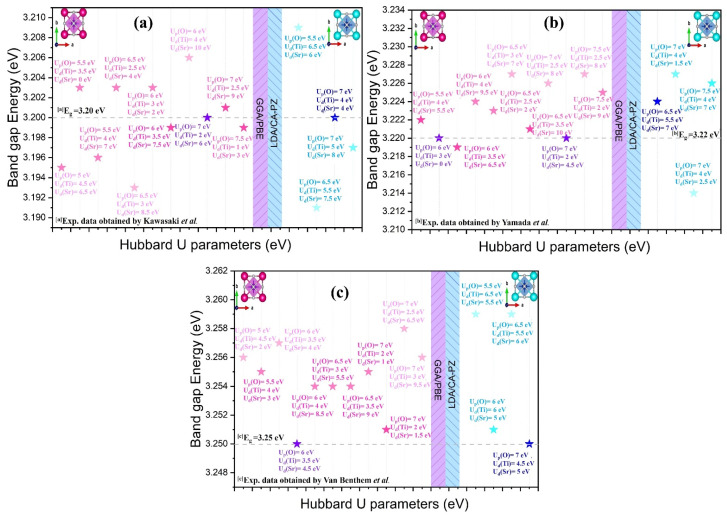
Comparison between the computed band gaps of the cubic phase of STO with experimental data highlighting the Hubbard U potential selected for the O-2p, Ti-3d, and Sr-4d orbitals using GGA/PBE and LDA/CA-PZ approximations. (**a**) the distinct colors of the Hubbard U potential for the O-2p, Ti-3d and Sr-4d electrons are respectively 7 eV, 2 eV and 6 eV for the GGA/PBE approximation and 7 eV, 4 eV and 4 eV for the LDA/CA-PZ approximation, resulting in a gap energy of 3.20 eV, in perfect agreement with the experimental value obtained by Kawasaki et al [11]. (**b**) the distinct colors of the Hubbard U potential for the O-2p, Ti-3d and Sr-4d electrons are respectively 7 eV, 2 eV and 4.5 eV for the GGA/PBE approximation and 6.5 eV, 5.5 eV and 7 eV for the LDA/CA-PZ approximation, resulting in a gap energy of 3.22 eV, in perfect agreement with the experimental value obtained by Yamada et al [12]. (**c**) the distinct colors of the Hubbard U potential for the O-2p, Ti-3d and Sr-4d electrons are respectively 6 eV, 3.5 eV and 4.5 eV for the GGA/PBE approximation and 7 eV, 4.5 eV and 5 eV for the LDA/CA-PZ approximation, resulting in a gap energy of 3.25 eV, in perfect agreement with the experimental value obtained by Van Benthem et al [13].

The overview presented in Table 3 and Figure 3 underlines the impact of the selected Hubbard U potential on the calculated band gaps by comparing them with those obtained experimentally. As can be seen in Table 3, we chose the Hubbard U potential of the cubic (*P*m$\bar{3}$m) phase of STO that exhibits minimal deviations. For the GGA/PBE approximation, we set the Hubbard U-potential for the O-2p, Ti-3d, and Sr-4d electrons to 7 eV, 2 eV, and 6 eV, respectively, giving gap energy of 3.200; to 7 eV, 2 eV, and 4.5 eV to achieve a gap energy of 3.220; and to 6 eV, 3.5 eV, and 4.5 eV to yield a gap energy of 3.250. For the LDA/CA-PZ approximation, we selected the Hubbard U-potential for the O-2p, Ti-3d, and Sr-4d electrons to 7 eV, 4 eV, and 4 eV, respectively, resulting in a gap energy of 3.200 as well as 6.5 eV, 5.5 eV, and 4 eV to achieve a gap energy of 3.224 and 7 eV, 4.57 eV, and 5 eV to obtain a gap energy of 3.250. The Hubbard potential previously stated, which gave us band-gap energies in perfect agreement with the experimental results [11,12,13], was used.

### 2.2. Electronic Properties

#### Band Structure and Density of States

**Figure 4:**
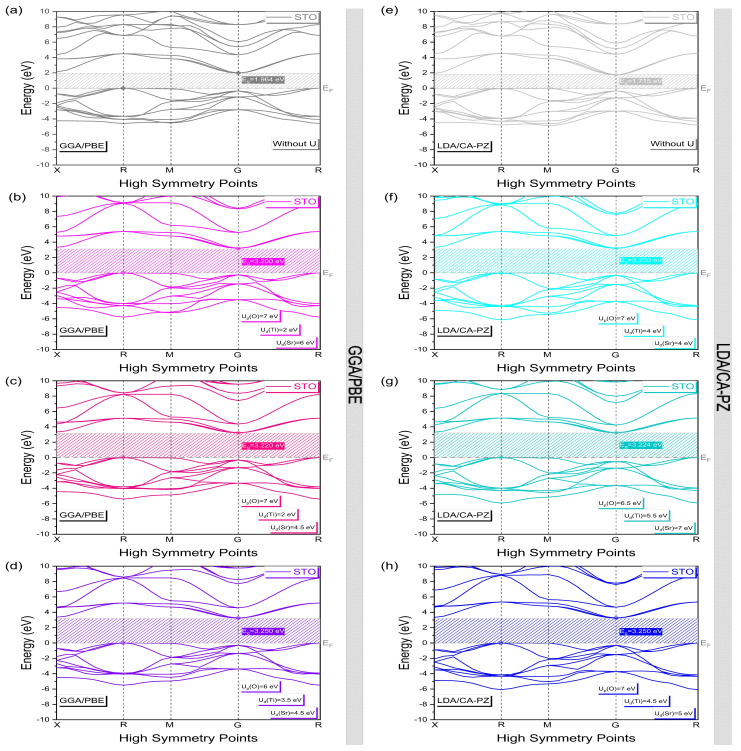
Band structure of the cubic phase (*P*m$\bar{3}$m) of STO with and without the Hubbard U correction, using (**a**–**d**) GGA/PBE approximation and (**e**–**h**) LDA/CA-PZ method. Each band’s energy is shifted with the Fermi level (EF) set to zero.

Figure 4 displays the computed band structures in the Brillouin zone (BZ) along high-symmetry directions for the cubic (*P*m$\bar{3}$m) phase of STO using the GGA/PBE and LDA/CA-PZ approximations with and without the Hubbard U correction. At the same time, Table 4 presents the calculated band gaps and the earlier theoretical and experimental results. When only using the GGA/PBE and LDA/CA-PZ approximations, as shown in Figure 4a,e, the band structure of the cubic (*P*m$\bar{3}$m) phase of STO has an indirect band gap of 1.964 eV and 1.715 eV at the R-G and M-G points, respectively. These computational findings are in agreement with those obtained theoretically (Table 4) by authors [13,20,21,22,31,34] using DFT and exchange-correlation functions, which provides an erroneous assessment of the band structure as well as could underestimate the measured band-gap values (3.20 eV [11], 3.22 eV [12], and 3.25 eV [13]). The Hubbard U correction should thus be investigated in our calculations to overcome the limitations of the traditional DFT approaches (GGA/PBE and LDA/CA-PZ). This enables us to obtain band gaps (Figure 4b–d,f–h) in reasonably good agreement with the actual data [11,12,13]. As a result, our calculations following the optimization and selection of the Hubbard potential given above for the cubic phase (*P*m$\bar{3}$m) of STO show the accuracy of our model.

**Figure 5:**
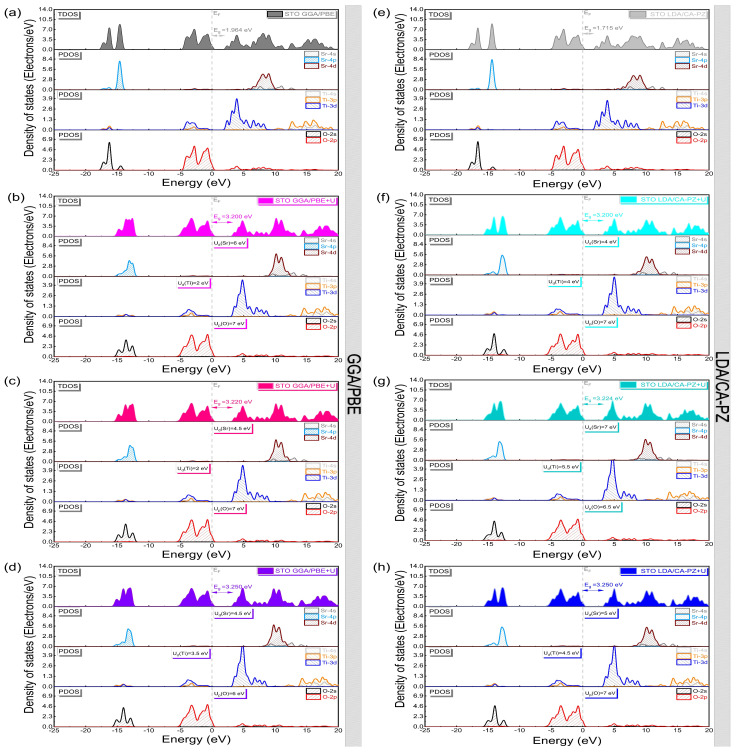
Total and partial density of states of the cubic phase (*P*m$\bar{3}$m) of STO with and without the Hubbard U correction, using (**a**–**d**) GGA/PBE approximation and (**e**–**h**) LDA/CA-PZ method.

**Figure 6:**
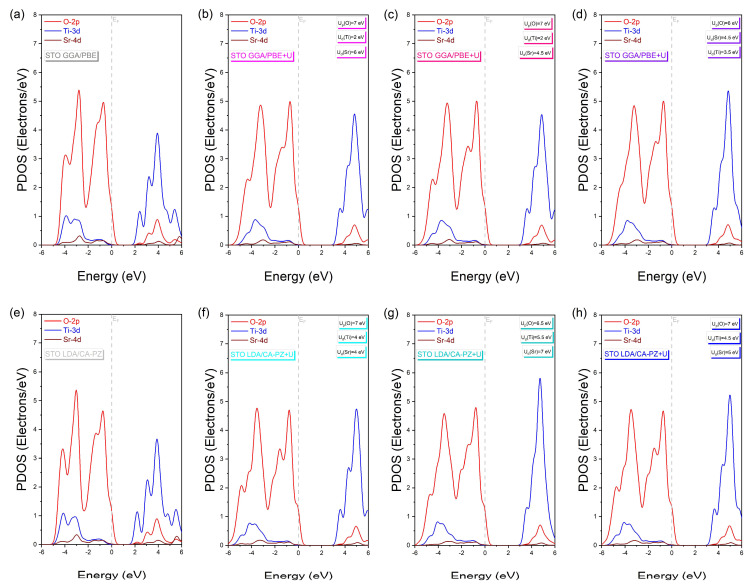
Partial densities of states of the cubic phase (*P*m$\bar{3}$m) of STO with and without the Hubbard U correction in the range between −6 eV and 6 eV, using (**a**–**d**) GGA/PBE approximation and (**e**–**h**) LDA/CA-PZ method.

We calculated the total (TDOS) and partial (PDOS) densities of states of the cubic (*P*m$\bar{3}$m) phase of STO using the two approximations to better understand the electronic properties, particularly the atomic orbital contributions to the formation of each energy band (Figure 5 and Figure 6).

With and without the Hubbard correction, the PDOS analysis shows the same electronic contributions for GGA/PBE and LDA/CA-PZ approximations (Figure 5a–h), where the valence band (VB) is mainly formed by contributions from the 2p and 2s oxygen orbitals and the 4p strontium orbitals, with a small contribution from the 3d and 4d titanium and strontium orbitals, respectively. On the other hand, the 3d orbital of titanium and the 4d orbital of strontium make up most of the conduction band (CB), with the 2p and 4s orbitals of oxygen and strontium, respectively, making up the remainder. In addition, at higher energies, the 3p and 4s titanium orbitals contribute to the conduction bands. Furthermore, as shown in the PDOS examination in Figure 6, the O-2p orbitals with Ti-3d hybridization characteristics predominate in the higher valence bands surrounding the Fermi level, reflecting the covalent character of the bonds between the Ti and O atoms. The decreased hybridization between the Sr and O atoms shows the ionic character.

**Figure 7:**
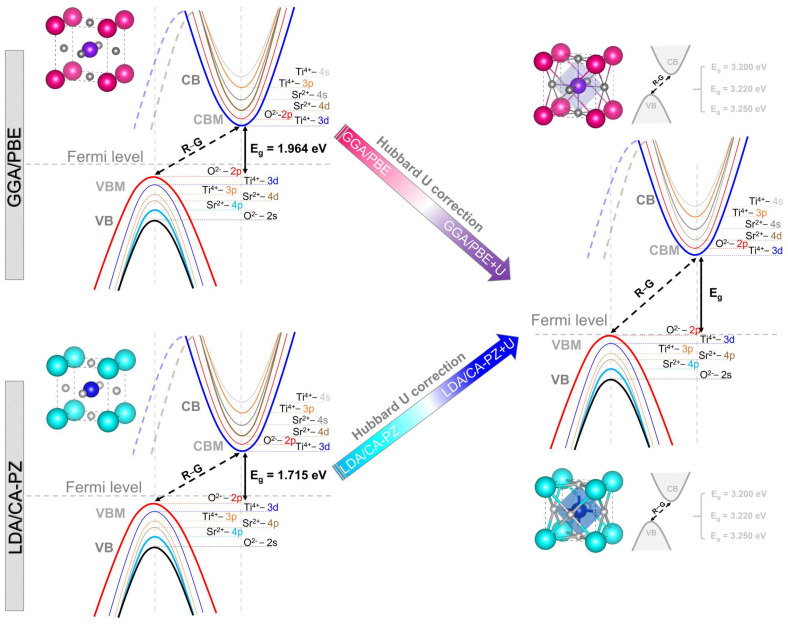
Schematic description of the effect of the Hubbard U correction on the orbital distribution of the cubic phase (*P*m$\bar{3}$m) of STO using the GGA/PBE and LDA/CA-PZ approximations.

In Figure 7, we present a description of the influence of the Hubbard U-correction on the orbital distribution using the partial densities of states to provide a deeper understanding of electronic distribution. We can observe that for both approximations (GGA/PBE and LDA/CA-PZ), similar electronic contributions are present, in which the upper part of the VBs comes mainly from O-2p orbitals. In contrast, the lower CBs come mainly from Ti-3d orbitals with the E_F_ fixed at 0 eV. Alternatively, in the case of the GGA/PBE (LDA/CA-PZ) approximation, the application of the Hubbard U-potential correction produces an increase in the intensity of the 3d titanium orbital, accompanied by a shift of 1.236 eV (1.485 eV), 1.256 eV (1.505 eV), and 1.286 eV (1.535 eV) of CB to higher energies, restoring the experimental band gaps of the cubic phase (*P*m$\bar{3}$m) of STO to 3.20 eV [11], 3.22 eV [12], and 3.25 eV [13], respectively. Hence, applying Hubbard correction, our computed electronic data pretty much followed the experimental data and compensated for the systematic error of the GGA/PBE and LDA/CA-PZ approximations.

### 2.3. Chemical Bonds

#### 2.3.1. Electron Charges Density

**Figure 8:**
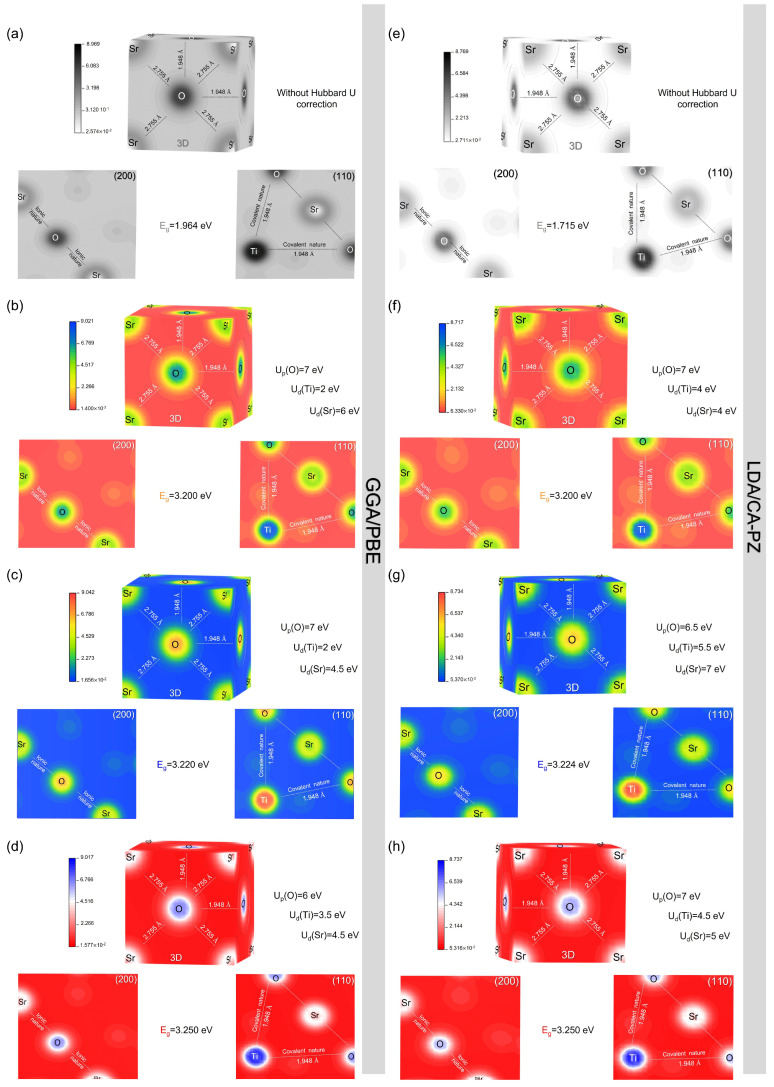
Electron density distribution maps in (3d) and (2d) in the relevant crystallographic planes: (200) and (110) for the cubic phase (*P*m$\bar{3}$m) of STO. (**a**,**e**) without Hubbard U correction using GGA/PBE and LDA/CA-PZ approximations, respectively. (**b**,**c**,**d**) with the Hubbard U correction for a gap energy of 3.200, 3.220 and 3.250, respectively, using GGA/PBE approximation. (**f**,**g**,**h**) with the Hubbard’s U correction for a gap energy of 3.200, 3.224 and 3.250, respectively, using LDA/CA-PZ approximation.

The electron density distribution maps were graphed in the relevant crystallographic planes of (200) and (110) in three (3d) and two (2d) dimensions for the cubic (*P*m$\bar{3}$m) phase of STO, using the GGA/PBE and LDA/CA-PZ approximations with and without Hubbard’s U potential, as illustrated in Figure 8a–h. The scales on the left side of each figure display the concentration of the electron density between Sr, Ti, and O ions, reflecting the strong electron density associated with high values, whereas, with decreasing values, there is a progressive disappearance of the electron distribution. In the (110) plane, electron density distributions between Ti and O exhibit electron overlap, indicating the covalent bond between the oxygen and titanium atoms (Figure 8), both with and without the Hubbard U correction. Additionally, in the (200) plane, strontium and oxygen’s electron distributions do not overlap, suggesting an ionic nature. These results support the covalent and ionic bonding agreements between Ti-O and Sr-O, respectively, that were previously described and are compatible with the body of literature [29,31,35,36] (Figure 6). Hence, this would make it possible to highlight the potential advantages of the STO material compared with other materials [37,38,39].

#### 2.3.2. Bond Lengths, Population, Mulliken Charges, and Effective Valence Charges

To determine the character of Sr-O and Ti-O bonds and to confirm the validity of our theoretical computations based on previous theoretical and experimental studies, the calculated bond lengths, population, Mulliken charges, and effective valence charges of the cubic (*P*m$\bar{3}$m) phase of STO by using the GGA/PBE+U and LDA/CA-PZ+U approximations are presented in Table 5. Based on Table 5 coupled with Figure 8, the optimized distances of Sr-O and Ti-O using only the GGA/PBE (LDA/CA-PZ) approximation are 2.778 Å (2.748 Å) and 1.964 Å (1.943 Å), respectively. After applying the Hubbard U correction, there is only a slight variation from the GGA/PBE and LDA/CA-PZ approximations in the optimized bond lengths. These findings are consistent with the theoretical computations obtained by Wei et al., whose values are 2.761 Å and 1.953 Å for the Sr–O and Ti–O bond distances, respectively [40], as well as those obtained by Li et al., whose values are 2.974 Å and 1.976 Å for the Sr–O and Ti–O bond distances, respectively [41]. Other than that, the Mulliken charges in such a crystal lattice reflect the significance of electron density sheared off by an atom, with most positive values indicating that the atom concerned contributes more electrons. 

The effective valence is the difference between the formal and effective ionic charges. Any perfectly ionic bond indicates that the value of the effective valence is zero, while raising the covalency means that the values are far from zero. As we can see from Table 5, without the Hubbard U correction for the GGA/PBE (LDA/CA-PZ) approximation, the calculated effective valence charges are 2.24e (2.17e) and 0.57e (0.65e) for Ti and Sr cations, respectively, indicating that the Ti-O and Sr-O bonds are covalent and ionic, respectively. After the application of the Hubbard U correction, a slight variation versus the classical DFT methods is observed in the practical valence charge values of Ti and Sr cations while keeping the covalent and ionic characteristics. These results confirm the accuracy of our earlier PDOS results (Figure 6) together with the electron charge density (Figure 8).

## 3. Computational Model

All computations were performed using plane-wave density functional theory simulations implemented in the CASTEP (Cambridge Serial Total Energy Package) code [42]. The exchange and correlation energies of the electrons were calculated using different exchange-correlation functions (ECFs) by using the generalized gradient approximation (GGA/PBE) [43,44] and the local density approximation (LDA/CA-PZ) [45,46], respectively. Additionally, several pseudopotentials’ approaches were employed to characterize the interactions between the valence electrons and ionic core for the valence electron configurations of 4s^2^4p^6^5s^2^ for Sr, 3s^2^3p^6^3d^2^4s^2^ for Ti and 2s^2^2p^4^ for O. Furthermore, the Brillouin zone integrations produced automatically using the Monkhorst–Pack technique [47] were carried out over a varied grid size to acquire the equilibrium lattice parameters with minimal deviations from the experiment. Then, using the GGA/PBE and LDA/CA-PZ approximations, the electron wave functions were extended through a plane wave basis set with different cut-off energies. Each calculation was considered convergent when the maximum root-mean-square convergent tolerance was 10^−5^ eV/atom and for maximum stress, maximum force, and maximum displacement, which are 0.05 GPa, 0.03 eV/Å, and 0.001 Å, respectively.

In the geometry optimization section, the most appropriate parameters used in geometric optimization to obtain the equilibrium lattice parameters with minimal deviations from the experimental results are as follows: for GGA/PBE, the pseudopotential method is OTGF ultrasoft, the cut-off energy is 800 eV, and the k-points are 2 × 2 × 2; for LDA/CA-PZ, the pseudopotential method is OTFG norm-conserving, the cut-off energy is 570 eV, and the k-points are 3 × 3 × 3. 

## 4. Conclusions

Detailed computations of the crystal structure, electronic properties, and chemical bands of the cubic phase (*P*m$\bar{3}$m) of STO with a detailed optimization were discussed. These findings were obtained by first-principles calculations based on DFT using the GGA/PBE and LDA/CA-PZ approximations and by applying Hubbard’s U-potential correction. A minimal variation (less than 1%) of the optimal lattice parameters/volume from data collected from experiments was produced by using the most appropriate plane wave pseudopotential methods, k-points, and cut-off energy, proving the reliability of our model. In addition, we also demonstrated from electronic studies that the Hubbard U-potential correction can overcome the underestimation induced by the GGA/PBE and LDA/CA-PZ approximations. Indeed, for the two approximations, the Hubbard U-potential values selected for the Ti-3d, Sr-4d, and O-2p orbitals led to a shift of CB to higher energies, providing band gaps in reasonably good agreement with the most common experimental band gaps (i.e., which are 3.20 eV, 3.24 eV, and 3.25 eV) of the cubic (*P*m$\bar{3}$m) phase of STO. Furthermore, PDOS calculations associated with the chemical band analysis suggest that the Sr-O bonds are predominantly ionic, while the Ti-O bonds are covalent, agreeing with previous theoretical investigations. As a result, our study demonstrates that DFT+U may be a helpful technique for obtaining accurate band-gap predictions at a reasonable computing cost while also making up for the systematic inaccuracy of approximations such as GGA/PBE and LDA/CA-PZ. In this context, findings may potentially be used as a theoretical guide for further studies on the cubic phase (*P*m$\bar{3}$m) of STO perovskite.

## Figures and Tables

**Table 1:** Appropriate k-points and cut-off energy values of the cubic (*P*m$\bar{3}$m) phase of STO using GGA/PBE and LDA/CA-PZ approximations.

Methods	K-Points	Cut-Off (eV)	a_i_ = b_i_ = c_i_ (Å)	a_f_ = b_f_ = c_f_ (Å)	Deviation (%)	V_i_ (Å^3^)	V_f_ (Å^3^)	Deviation (%)
STO: (*P*m$\bar{3}$m) [Pseudopotential methods: OTFG Ultrasoft (GGA/PBE), OTFG norm-conserving (LDA/CA-PZ)]								
GGA/PBE	2 × 2 × 2	800	3.901 ^[a]^	3.9297	0.7303	59.365 ^[a]^	60.687	2.1783
LDA/CA-PZ	3 × 3 × 3	570	3.901 ^[a]^	3.9005	−0.0128	59.365 ^[a]^	59.344	−0.0353

^[a]^ Experimental data from Ref. [29]; a_i_ and a_f_ are experimental and optimized lattice parameters, respectively. V_i_ and V_f_ are experimental and optimized volume, respectively.

**Table 2:** The optimized lattice constants (a = b = c in Å) using the GGA-PBE and LDA/CA-PZ approximations, in comparison with available theoretical and experimental data of the cubic (*P*m$\bar{3}$m) phase of STO.

Structure	This Calculation		Experimental Data	Previous Theoretical Results (DFT Functional)
	DFT Functional	a = b = c		
STO	GGA/PBE	3.9297	3.901 [29], 3.907 [20].	3.850 (LDA) [20], 3.86 (LDA), 3.95 (PWGGA), 3.94 (PBE), 3.98 (BLYP), 3.90 (P3PW), 3.94 (P3LYP), 3.92 (HF) [31], 3.862 (LSDA), 3.947 (PBE-GGA) [32].

**Table 3:** Selected Hubbard U potential for the O-2p, Ti-3d, and Sr-4d orbitals of the cubic phase (*P*m$\bar{3}$m) of STO using the GGA/PBE and LDA/CA-PZ approximations. In our calculations, the cut-off energy/k-points values were set at 800 eV/2 × 2 × 2 and 570 eV/3 × 3 × 3 for the GGA/PBE and LDA/CA-PZ approximations, respectively.

Methods	U_p_(O)	U_d_(Ti)	U_d_(Sr)	E_g_ (eV)		Deviations (%)
				This Cal.	Exp Data.	
GGA/PBE	**0**	**0**	**0**	**1.964**	**-**	**-**
	5	4.5	6.5	3.195	3.20 [11]	−0.156
	5.5	3.5	0	3.203		0.093
	5.5	4	7	3.196		−0.125
	6.5	2.5	4	3.203		0.093
	6.5	3	8.5	3.193		−0.219
	6	3	2	3.203		0.093
	6	3.5	7.5	3.199		−0.031
	6	4	10	3.206		0.187
	**7**	**2**	**6**	**3.200**		**0**
	7	2.5	9	3.201		0.031
	7.5	1	3	3.199		−0.031
	5.5	4	5.5	3.222	3.22 [12]	0.062
	6	3	0	3.220		0
	6	3.5	6.5	3.219		−0.031
	6	4	9.5	3.224		0.124
	6.5	2.5	2	3.223		0.093
	6.5	3	7	3.227		0.216
	6.5	3.5	10	3.221		0.031
	7	2	4	3.226		0.185
	**7**	**2**	**4.5**	**3.220**		**0**
	7	2.5	8	3.227		0.216
	7.5	2	9	3.225		0.155
	5	4.5	2	3.256	3.25 [13]	0.184
	5.5	4	3	3.255		0.153
	6	3.5	4	3.257		0.214
	**6**	**3.5**	**4.5**	**3.250**		**0**
	6	4	8.5	3.254		0.122
	6.5	3	5.5	3.254		0.122
	6.5	3.5	9	3.254		0.122
	7	2	1	3.255		0.153
	7	2	1.5	3.251		0.030
	7	2,5	6.5	3.258		0.245
	7	3	9.5	3.256		0.184
LDA/CA-PZ	**0**	**0**	**0**	**1.715**	**-**	**-**
	5.5	6.5	6	3.209	3.20 [11]	0.280
	6.5	5.5	7.5	3.191		−0.282
	**7**	**4**	**4**	**3.200**		**0**
	7	5	8	3.197		−0.093
	**6.5**	**5.5**	**7**	**3.224**	3.22 [12]	**0.124**
	7	4	1.5	3.227		0.216
	7	4	2.5	3.214		−0.186
	7.5	4	7	3.226		0.185
	5.5	6.5	5.5	3.259	3.25 [13]	0.276
	6	6	5	3.251		0.030
	6.5	5.5	6	3.259		0.276
	**7**	**4.5**	**5**	**3.250**		**0**

The bold numbers indicate the gap energy value obtained without using Hubbard’s potential (For GGA E_g_ = 1.964 eV; For LDA E_g_ = 1.715 eV).

**Table 4:** Computed indirect band gaps for the cubic (*P*m$\bar{3}$m) phase of STO, using GGA/PBE and LDA/CA-PZ approximations, with and without Hubbard U correction, as well as previous theoretical and experimental results.

These Calculations (eV)				Exp. Data (eV)	Previous Theoretical Results (eV) Indirect Band Gaps (Without U)
Without U		With U			
Methods		Methods			
GGA/PBE	LDA/CA-PZ	GGA/PBE	LDA/CA-PZ		
1.964 [R-G]	1.715 [M-G]	3.200 [R-G]	3.200 [R-G]	3.20 [11]	1.89 [R-Γ] (LDA) [13], 1.90 [R-Γ] (LDA) [20], 1.79 [R-Γ] (LDA PW) [21], 1.73 (GGA) [22], 2.04 [R-Γ] (LDA), 1.97 [R-Γ] (PWGGA), 1.99 [R-Γ] (PBE) [31], 1.8 (LCAO/PBE), 1.8 (PW/PBE) [34].
		3.220 [R-G]	3.224 [R-G]	3.22 [12]	
		3.250 [R-G]	3.250 [R-G]	3.25 [13]	

**Table 5:** Calculated Mulliken charges, effective valence charges, population, and bond lengths of the cubic (*P*m$\bar{3}$m) phase of STO by using the GGA/PBE+U and LDA/CA-PZ+U approximations.

$\mathrm{STO}\;[\mathrm{Cubic};\;Pm\bar{3}$$m;\;O_{h}^{1}$]									
Method	Species	Mulliken Charges (e)				Effective Valence Charges (e)			
		E_g_ = 1.964 (eV)	E_g_ = 3.200 (eV)	E_g_ = 3.220 (eV)	E_g_ = 3.250 (eV)	E_g_ = 1.964 (eV)	E_g_ = 3.200 (eV)	E_g_ = 3.220 (eV)	E_g_ = 3.250 (eV)
GGA/PBE	Sr	1.37	1.61	1.58	1.56	0.57	0.36	0.39	0.40
	Ti	0.85	1.02	1.03	1.03	2.24	1.93	1.93	1.92
	O_1_=O_2_=O_3_	−0.74	−0.88	−0.87	−0.87	0.00	0.00	0.00	0.00
Method	Species	Mulliken charges (e)				Effective valence charges (e)			
		E_g_ = 1.715 (eV)	E_g_ = 3.200 (eV)	E_g_ = 3.224 (eV)	E_g_ = 3.250 (eV)	E_g_ = 1.715 (eV)	E_g_ = 3.200 (eV)	E_g_ = 3.224 (eV)	E_g_ = 3.250 (eV)
LDA/CA-PZ	Sr	1.35	1.57	1.66	1.60	0.65	0.45	0.34	0.42
	Ti	0.84	1.06	1.06	1.07	2.17	1.80	1.76	1.78
	O_1_=O_2_=O_3_	−0.73	−0.88	−0.90	−0.89	0.00	0.00	0.00	0.00
Method	Bond	Population				Lengths (Å)			
		E_g_ = 1.964 (eV)	E_g_ = 3.200 (eV)	E_g_ = 3.220 (eV)	E_g_ = 3.250 (eV)	E_g_ = 1.964 (eV)	E_g_ = 3.200 (eV)	E_g_ = 3.220 (eV)	E_g_ = 3.250 (eV)
GGA/PBE	O-Sr	0.13	0.04	0.05	0.06	2.778	2.791	2.782	2.792
	O-Ti	0.86	0.92	0.92	0.91	1.964	1.973	1.967	1.974
	O-O	−0.14	−0.13	−0.13	−0.13	2.778	2.791	2.782	2.792
Method	Bond	Population				Lengths (Å)			
		E_g_ = 1.715 (eV)	E_g_ = 3.200 (eV)	E_g_ = 3.224 (eV)	E_g_ = 3.250 (eV)	E_g_ = 1.715 (eV)	E_g_ = 3.200 (eV)	E_g_ = 3.224 (eV)	E_g_ = 3.250 (eV)
LDA/CA-PZ	O-Sr	0.10	0.03	0.01	0.02	2.748	2.760	2.797	2.773
	O-Ti	0.90	0.96	0.98	0.97	1.943	1.952	1.978	1.960
	O-O	−0.16	−0.15	−0.14	−0.14	2.748	2.760	2.797	2.773

## Data Availability

The data presented in this study are available from the corresponding author upon reasonable request.

## Supplementary Materials

The following supporting information can be downloaded at: https://www.mdpi.com/article/10.3390/molecules29133081/s1, Table S1: Computation of the cell parameters and volume deviation using the GGA/PBE and LDA/CA-PZ approximations, of cubic phase (*P*m$\bar{3}$m) of STO as a function of the variation of the pseudopotential methods; The k-points and cut-off energy values were fixed at 2 × 2 × 2 and 500 eV, respectively; Figure S1: Total and partial density of states of the cubic phase (*P*m$\bar{3}$m) of STO via GGA/PBE approximation by using: (**a**) the OTGF ultrasoft pseudopotential method and (**b**) the OTGF norm-conserving pseudopotential method; Table S2: Computation of the cell parameters and volume deviation using the GGA/PBE and LDA/CA-PZ approximations, of cubic phase (*P*m$\bar{3}$m) of STO as a function of the variation of the k-points values; The value of cut-off energy was set at 500 eV; Table S3: Computation of the cell parameters and volume deviation using the GGA/PBE and LDA/CA-PZ approximations, of cubic phase (*P*m$\bar{3}$m) of STO as a function of the variation of the cut-off energy values; The k-points values were set at 2 × 2 × 2 for the GGA/PBE approximation and 3 × 3 × 3 for the LDA/CA-PZ approximation.
