# Supplementary material for: Influence of the Hubbard U Correction on the Electronic Properties and Chemical Bands of the Cubic (Pm3¯m) Phase of SrTiO3 Using GGA/PBE and LDA/CA-PZ Approximations
Source: Molecules. 2024 Jun 28;29(13):3081. doi: 10.3390/molecules29133081 (PMC11243698; doi:10.3390/molecules29133081)
Supplement: Supplementary file 1 [file molecules-29-03081-s001.zip › molecules-3041699-supplementary.pdf]

Supplementary Materials for

Influence of the Hubbard U Correction on the Electronic Properties and Chemical Bands of the Cubic (*P*m$\overline{3}$m) Phase of $SrTiO_3$ Using GGA/PBE and LDA/CA-PZ Approximations

Issam Derkaoui [1], Mohamed Achehboune [2], Roberts I. Eglitis [3], Anatoli I. Popov [3], Issam Boukhoubza [1], Mohamed A. Basyooni-M. Kabatas [4,5,6,*] and Abdellah Rezzouk [1]

[1] Laboratory of Solid State Physics, Faculty of Sciences Dhar el Mahraz, University Sidi Mohammed Ben Abdellah, P.O. Box 1796, Atlas Fez 30 000, Morocco; derkaouiissam@gmail.com (I.D.)

[2] Laboratoire de Physique des Solides, Namur Institute of Structured Matter, University of Namur, Rue de Bruxelles 61, 5000 Namur, Belgium

[3] Institute of Solid State Physics, University of Latvia, 8 Kengaraga Str., LV1063 Riga, Latvia

[4] Dynamics of Micro and Nano Systems Group, Department of Precision and Microsystems Engineering, Delft University of Technology, Mekelweg 2, 2628 CD Delft, The Netherlands

[5] Department of Nanotechnology and Advanced Materials, Graduate School of Applied and Natural Science, Selçuk University, Konya 42030, Turkey

[6] Solar Research Laboratory, Solar and Space Research Department, National Research Institute of Astronomy and Geophysics, Cairo 11421, Egypt

* Correspondence: m.kabatas@tudelft.nl or m.a.basyooni@gmail.com

Contents

1. Geometry optimization …………………………………………………………………..…………...……. ii

1.1 Appropriate pseudopotential methods ……………………………………………………………. ii

1.2 Appropriate k-points and cut-off energy ……………………………………………...…….. iii

3. References ………………………………………….…….………………...…….…...…….……...……. v

1. Geometry Optimization

The following simulations were carried out under different plane-wave pseudopotential methods, using the GGA/PBE [1,2] and LDA/CA-PZ [3,4] approximations as implemented in the CASTEP code [5], respectively.

1.1 Appropriate pseudopotential methods

The lattice parameters and volume calculated by the different DFT functionals in this work compared with the experimental results for the cubic ($Pm\bar{3}m$) phase of STO perovskite materials, are listed in Tables S1, S2 and S3. At first, we will search for the most appropriate plane-wave pseudopotential methods (the ultrasoft, the OTGF ultrasoft and the OTGF norm-conserving) for the cubic phase ($Pm\bar{3}m$) of STO (see Table S1). Initially, the primitive cell parameters a = b = c = 3.901 Å (α = 90° ; volume = 59.365 Å) [6] were used for constructing the cubic phase ($Pm\bar{3}m$) of STO.

Table S1. Computation of the cell parameters and volume deviation using the GGA/PBE and LDA/CA-PZ approximations, of cubic phase ($Pm\bar{3}m$) of STO as a function of the variation of the pseudopotential methods; The k-points and cut-off energy values were fixed at 2×2×2 and 500 eV, respectively.

Methods	Pseudopotential methods	a_i=b_i=c_i (Å)	a_f=b_f=c_f (Å)	Deviation (%)	V_i (Å^3)	V_f (Å^3)	Deviation (%)
**STO**: ($Pm\bar{3}m$) [**k-points**: 2×2×2; **Cut-off**: 500 eV]							
GGA/PBE	Ultrasoft	3.901[a]	3.9443	1.0977	59.365[a]	61.366	3.2607
	**OTFG ultrasoft**		3.9406	**1.0049**		61.193	**2.9872**
	OTFG norm-conserving		3.9224	0.5455		60.347	1.6289
LDA/CA-PZ	Ultrasoft	3.901[a]	3.8518	-1.2773	59.365[a]	57.150	-3.8740
	OTFG ultrasoft		3.8567	-1.1486		57.365	-3.4847
	**OTFG norm-conserving**		3.8574	**-1.1302**		57.398	**-3.4252**

[a]Experimental data from Ref. [6]
a_i and a_f experimental and optimized lattice parameters, respectively.
V_i and V_f experimental and optimized volume, respectively.

For the STO cubic phase, when compared with the same experimental values obtained by Abramov et al. [6], the relative deviations between the calculated structure parameters and the experimental values for the GGA/PBE (LDA/CA-PZ) approximation are about 1.097% (-1.277%) for the ultrasoft, 1.004% (-1.148%) for the OTGF ultrasoft and 0.545% (-1.130%) for the OTGF norm-conserving pseudopotential methods. For GGA approximation, it should be noted that the OTGF norm-conserving pseudopotential,

even if it offers better deviations in the mesh and volume parameters than the OTGF ultrasoft pseudopotential, but it causes a problem related to the disappearance of the Sr-4d orbital in the density of states. For this reason, we have chosen the OTGF ultrasoft pseudopotential and for a better understanding we present the two partial densities of electronic states of the two potentials (Figure S1). Therefore, the lattice parameters for STO calculated by OTGF ultrasoft (GGA/PBE) and OTFG norm-conserving (LDA/CA-PZ) pseudopotential methods have the smallest relative deviation from the experimental value.

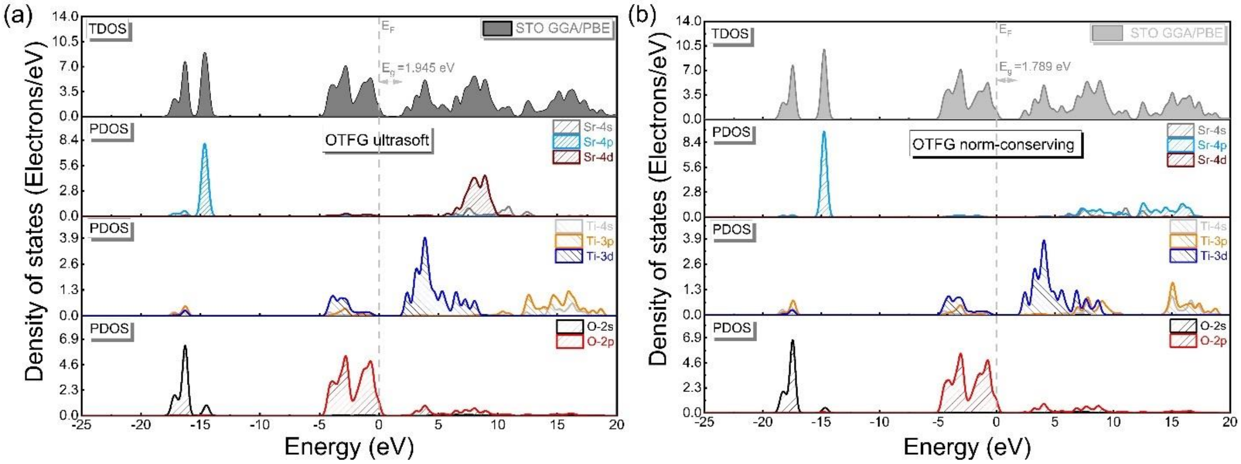

Figure. S1. Total and partial density of states of the cubic phase ($Pm\overline{3}m$) of STO via GGA/PBE approximation by using: **(a)** the OTGF ultrasoft pseudopotential method and **(b)** the OTGF norm-conserving pseudopotential method.

1.2 Appropriate k-points and cut-off energy

After finding the most suitable pseudopotential methods for STO, we shall then search for the most suitable k-points and cut-off energy values. As a preliminary step for both the GGA/PBE and LDA/CA-PZ approximations, the energy cut-off value will be kept constant (e.g., 500 eV) and the grid values (k-points) will be varied (see Table S2). Once we have obtained an appropriate value for the k-points, we will hold this value constant and modify the cut-off energy to achieve the most appropriate cut-off energy value (see Table S3).

Table S2. Computation of the cell parameters and volume deviation using the GGA/PBE and LDA/CA-PZ approximations, of cubic phase (*P*m$\overline{3}$m) of STO as a function of the variation of the k-points values; The value of cut-off energy was set at 500 eV.

Methods	k-points	$a_i=b_i=c_i$ (Å)	$a_f=b_f=c_f$ (Å)	Deviation (%)	V_i (Å^3)	V_f (Å^3)	Deviation (%)
**STO**: (*P*m$\overline{3}$m) [**Pseudopotential methods**: OTFG Ultrasoft (*GGA/PBE*), OTFG norm-conserving (*LDA/CA-PZ*)]							
GGA/PBE	1×1×1	3.901[a]	failed	-	59.365[a]	failed	-
	**2×2×2**	3.901[a]	3.9406	**1.0049**	59.365[a]	61.086	**2.8189**
	3×3×3	~	3.9484	1.2004	~	61.555	3.5577
	4×4×4	~	3.9443	1.0977	~	61.366	3.2607
	5×5×5	~	3.9466	1.1554	~	61.474	3.4307
	6×6×6	~	3.9443	1.0977	~	61.366	3.2607
	7×7×7	~	3.9452	1.1203	~	61.408	3.3269
	8×8×8	~	3.9442	1.0952	~	61.363	3.2560
	9×9×9	3.901[a]	3.9453	1.1228	59.365[a]	61.412	3.3332
LDA/CA-PZ	1×1×1	~	failed	-	~	failed	-
	2×2×2	~	3.8574	-1.1303	~	57.397	-3.4287
	**3×3×3**	3.901[a]	3.8972	**-0.0975**	59.365[a]	59.193	**-0.2905**
	4×4×4	~	3.8706	-0.7854	~	57.992	-2.3675
	5×5×5	~	3.8793	-0.5593	~	58.383	-1.6819
	6×6×6	~	3.8733	-0.7151	~	58.110	-2.1596
	7×7×7	~	3.8796	-0.5516	~	58.394	-1.6628
	8×8×8	~	3.8721	-0.7463	~	58.057	-2.2529
	9×9×9	3.901[a]	3.8767	-0.6268	59.365[a]	58.265	-1.8879

The minimum deviation from the experimental and optimized volume and lattice parameters is when the k-points are fixed at 2×2×2 and 3×3×3 using the GGA/PBE and LDA/CA-PZ approximations, respectively (Table S2). Furthermore, we have found that after applying several cut-off energies, the minimum deviation of the lattice parameters and volume is 0.730% (-0.012%) and 2.178% (-0.035%) using the GGA/PBE (LDA/CA-PZ) approximation, respectively, and this is for the cut-off energy value of 800 eV (570 eV) under the GGA/PBE (LDA/CA-PZ) approximation, respectively (Table S3).

Accordingly, for STO perovskite material, these findings demonstrate that the deviation of lattice parameters between calculated and standard values [6] was less than 0.74% (0.013%), under the GGA/PBE (LDA/CA-PZ) approximation. Therefore, the cut-off and k-points values chosen after optimization are reliable and proves the validity of our model, simultaneously showing the lowest relative deviation from the experimental values. Thereby, our results, investigate the performance and diversity of structural parameters for STO calculated by the GGA/PBE and LDA/CA-PZ approximations.

Table S3. Computation of the cell parameters and volume deviation using the GGA/PBE and LDA/CA-PZ approximations, of cubic phase (*P*m$\overline{3}$m) of STO as a function of the variation of the cut-off energy values; The k-points values were set at 2×2×2 for the GGA/PBE approximation and 3×3×3 for the LDA/CA-PZ approximation.

**Methods**	**Cut-off (eV)**	**$a_i=b_i=c_i$ (Å)**	**$a_f=b_f=c_f$ (Å)**	**Deviation (%)**	**V_i (Å^3)**	**V_f (Å^3)**	**Deviation (%)**
**STO**: (*P*m$\overline{3}$m) [**Pseudopotential methods**: OTFG Ultrasoft (*GGA/PBE*), OTFG norm-conserving (*LDA/CA-PZ*)]							
GGA/PBE	200	3.901[a]	4.7326	17.5717	59.365[a]	105.998	43.9942
	300	~	3.9916	2.2697	~	63.601	6.6602
	400	~	3.9884	2.1913	~	63.445	6.4307
	500	~	3.9406	1.0049	~	61.086	2.8189
	600	~	3.9314	0.7732	~	60.766	2.3055
	700	~	3.9302	0.7429	~	60.710	2.2154
	780	~	3.9298	0.7328	~	60.692	2.1864
	790	~	3.9301	0.7404	~	60.707	2.2106
	**800**	3.901[a]	3.9297	**0.7303**	59.365[a]	60.687	**2.1783**
	810	~	3.9299	0.7353	~	60.694	2.1896
	820	~	3.9299	0.7353	~	60.695	2.1912
	830	~	3.9298	0.7328	~	60.689	2.1816
	840	~	3.9297	0.7303	~	60.689	2.1816
	860	~	3.9298	0.7328	~	60.689	2.1816
	880	~	3.9298	0.7328	~	60.692	2.1864
	900	3.901[a]	3.9301	0.7404	59.365[a]	60.704	2.2057
LDA/CA-PZ	200	3.901[a]	4.6214	15.5883	59.365[a]	98.706	39.8567
	300	~	3.9464	1.1504	~	61.465	3.4165
	400	~	4.0294	3.1865	~	65.423	94.0372
	500	~	3.8972	-0.0975	~	59.193	-0.2905
	520	~	3.8864	-0.3756	~	58.702	-1.1294
	540	~	3.9085	0.19188	~	59.709	59.709
	560	~	3.9067	0.1459	~	59.628	0.4410
	**570**	3.901[a]	3.9005	**-0.0128**	59.365[a]	59.344	**-0.0353**
	580	~	3.8977	-0.0846	~	59.216	-0.2516
	590	~	3.9130	0.3066	~	59.914	0.91631
	600	~	3.8793	-0.5593	~	58.382	-1.6837
	700	~	3.8611	-1.0333	~	57.562	-3.1322
	800	~	3.8677	-0.8609	~	57.858	-2.6046
	900	3.901[a]	3.8627	-0.9915	59.365[a]	57.636	-2.9998

References

[1] D. Vanderbilt, Soft self-consistent pseudopotentials in a generalized eigenvalue formalism, Phys. Rev. B. 41 (1990) 7892–7895. https://doi.org/10.1103/PhysRevB.41.7892.

[2] J.P. Perdew, K. Burke, M. Ernzerhof, Generalized Gradient Approximation Made Simple, Phys. Rev. Lett. 77 (1996) 3865–3868. https://doi.org/10.1103/PhysRevLett.77.3865.

[3] D.M. Ceperley, B.J. Alder, Ground State of the Electron Gas by a Stochastic Method, Phys. Rev. Lett. 45 (1980) 566–569. https://doi.org/10.1103/PhysRevLett.45.566.

[4] J.P. Perdew, A. Zunger, Self-interaction correction to density-functional approximations for many-electron systems, Phys. Rev. B. 23 (1981) 5048–5079. https://doi.org/10.1103/PhysRevB.23.5048.

[5] S.J. Clark, M.D. Segall, C.J. Pickard, P.J. Hasnip, M.I.J. Probert, K. Refson, M.C. Payne, First principles methods using CASTEP, Z. fur Krist. - Cryst. Mater. 220 (2005) 567–570. https://doi.org/10.1524/zkri.220.5.567.65075.

[6] Yu.A. Abramov, V.G. Tsirelson, V.E. Zavodnik, S.A. Ivanov, Brown I. D., The chemical bond and atomic displacements in $SrTiO_3$ from X-ray diffraction analysis, Acta Crystallogr B Struct Sci. 51 (1995) 942–951. https://doi.org/10.1107/S0108768195003752.
